# Human-Written vs AI-Generated Texts in Orthopedic Academic Literature: Comparative Qualitative Analysis

**DOI:** 10.2196/52164

**Published:** 2024-02-16

**Authors:** Hassan Tarek Hakam, Robert Prill, Lisa Korte, Bruno Lovreković, Marko Ostojić, Nikolai Ramadanov, Felix Muehlensiepen

**Affiliations:** 1 Center of Orthopaedics and Trauma Surgery University Clinic of Brandenburg Brandenburg Medical School Brandenburg an der Havel Germany; 2 Faculty of Health Sciences University Clinic of Brandenburg Brandenburg an der Havel Germany; 3 Center of Evidence Based Practice in Brandenburg, a JBI Affiliated Group Brandenburg an der Havel Germany; 4 Center of Health Services Research Faculty of Health Sciences University Clinic of Brandenburg Rüdersdorf bei Berlin Germany; 5 Faculty of Orthopaedics University Hospital Merkur Zagreb Croatia; 6 Departement of Orthopaedics University Hospital Mostar Mostar Bosnia and Herzegovina

**Keywords:** artificial intelligence, AI, large language model, LLM, research, orthopedic surgery, sports medicine, orthopedics, surgery, orthopedic, qualitative study, medical database, feedback, detection, tool, scientific integrity, study design

## Abstract

**Background:**

As large language models (LLMs) are becoming increasingly integrated into different aspects of health care, questions about the implications for medical academic literature have begun to emerge. Key aspects such as authenticity in academic writing are at stake with artificial intelligence (AI) generating highly linguistically accurate and grammatically sound texts.

**Objective:**

The objective of this study is to compare human-written with AI-generated scientific literature in orthopedics and sports medicine.

**Methods:**

Five original abstracts were selected from the PubMed database. These abstracts were subsequently rewritten with the assistance of 2 LLMs with different degrees of proficiency. Subsequently, researchers with varying degrees of expertise and with different areas of specialization were asked to rank the abstracts according to linguistic and methodological parameters. Finally, researchers had to classify the articles as AI generated or human written.

**Results:**

Neither the researchers nor the AI-detection software could successfully identify the AI-generated texts. Furthermore, the criteria previously suggested in the literature did not correlate with whether the researchers deemed a text to be AI generated or whether they judged the article correctly based on these parameters.

**Conclusions:**

The primary finding of this study was that researchers were unable to distinguish between LLM-generated and human-written texts. However, due to the small sample size, it is not possible to generalize the results of this study. As is the case with any tool used in academic research, the potential to cause harm can be mitigated by relying on the transparency and integrity of the researchers. With scientific integrity at stake, further research with a similar study design should be conducted to determine the magnitude of this issue.

## Introduction

Artificial intelligence (AI) is perhaps best defined as an algorithmic mechanism applied to machines, whereby solving challenges requires little to no human interaction [1]. Differentiating human-made and AI-generated work is becoming increasingly difficult with the rapid technological advancement of deep learning [2]. Deep learning is based on the replication of human thinking and the brain’s structure [3]. With the vast potential benefit that AI might bring to the table, extensive research has been conducted in the last decade with the purpose of finding potential solutions for health care–related problems [4]. The field of orthopedics, for example, might greatly benefit from AI image recognition capabilities to assist in the diagnosis of fractures or skin lesions. Other benefits can be drawn from AI’s capacity to analyze massive amounts of clinical information, which in turn presents benefits in clinical decision-making, risk assessment, and the generation of individualized care plans [5]. That is why an exponential increase in research on the topic of AI in the field of orthopedics has been noted, which has led to a subsequent increase in reviews trying to summarize the findings and give out recommendations [4].

Orthopedic sports medicine is the subspecialty of orthopedics that deals with pathologic conditions of the musculoskeletal system that arise from the practice of sports. This includes the prevention, diagnosis, and treatment of diseases. A particular challenge of sports medicine lies in the willingness of athletes to return to performance in a timely manner [4]. Through the use of deep neural networks, AI can assist specialists in various aspects of management. AI has shown to be especially advantageous for the diagnosis of fractures based on plain radiographs and computed tomography, with reviews reporting high accuracy, sensitivity, and specificity for the evaluation of plain radiographs [6] and computed tomography images [7]. With the evolution of convolutional neural networks and the increased capacity to integrate large amounts of written information, the patient’s medical records could serve as a basis for determining an individualized care plan as well as for making predictions for the best future course of treatment [8].

The influence of large language models (LLMs) on research in the field of orthopedics and sports medicine has not yet been well studied. AI is commonly used by researchers to help organize thought processes, obtain feedback, edit their work, and present their citations in the requested format. Consequently, AI has made academic work much more efficient [9]. However, considering that some of the most impactful journals allow the use of AI in composing or editing scientific texts, there are some ethical reservations regarding the authenticity and credibility of academic work [2]. Furthermore, some journals are actively involved in the development of tools to spot AI-generated texts [10]. In the light of this, the line where scientific research becomes fraudulent with regards to the use of AI must be determined. Different journals have adopted different guidelines for the use of AI.

The aim of this qualitative analysis is to determine the possibility that human researchers and AI-detection platforms can detect AI-generated texts. For this purpose, 4 researchers were recruited to participate in this study. As well as this, an AI-detection platform was used to assist in this endeavor.

## Methods

This study adopted a similar method to previously conducted research on the matter [10].

### Recruitment

For the purposes of the study, 4 participants were recruited. Two senior researchers in the fields of orthopedics and qualitative research, as well as 2 junior researchers in the same fields, expressed their interest in the subject at hand. All researchers were informed about the study’s objectives. The inclusion criteria for senior researchers were more than 10 years of research experience and having a doctoral degree in their field. Junior researchers were defined as students or physicians who had commenced their first project in the last 2 years.

### Ethical Considerations

Due to the noninterventional nature of this study, as well as the anonymization of the included participants, local institutional and regulatory bodies did not require ethical approval. The methodology of the study and data collection were in line with the Geneva conventions. Informed consent was obtained from all participants involved in this study. The privacy and confidentiality of the involved participants has been protected by anonymizing their responses. No compensation was given to the participating individuals.

### Selection of Literature

After searching PubMed for relevant material, 5 abstracts about meniscal injuries were selected for inclusion in the study [11-15]. The search strategy included the word “meniscus.” Subsequently, the first 5 articles published in reputable first quartile (Q1) or second quartile (Q2) journals were chosen to ensure the high quality of the articles. Abstracts that did not meet the criteria were excluded. This choice was made based on the fact that abstracts usually present a general overview of the topic at hand and communicate the main objectives of the paper. Although some treatment modalities are commonly applied to meniscal injuries, it is often impossible to completely restore the meniscal architecture, especially when the injury occurs in the middle, less vascularized portion [16]. Selecting meniscal injuries as a topic was, therefore, agreed upon by the research team as it is a common pathologic condition [17] and an area of extensive research [18].

### Involving AI

Abstracts selected in the previous step were then rewritten by 2 AI platforms. One platform was the commonly used and extensively developed ChatGPT 3.4 (OpenAI) and the other was You.com. Using the instruction “rewrite the following in perfect academic English,” 5 new abstracts were generated by each AI. In the subsequent step, the command “write five abstracts on meniscal injuries” was used and 10 further abstracts were generated.

### Randomization

The 25 resulting abstracts included the 5 original versions that were written by humans, the 5 rewritten versions that were generated by each AI, and the 5 newly generated versions that were composed by each AI platform. The abstracts were numbered from 1 to 25. These numbers were subsequently randomized using Microsoft Excel and the assigned abstracts were presented as a sheaf in the resulting order.

### Evaluation

Evaluation of the abstracts was carried out using 2 methods. The first method of evaluation involved researchers with varying specialties and at different stages of their academic careers, while the second was based on the use of AI-detection software.

Participants were then asked to evaluate all the resulting abstracts using parameters that are commonly used for peer review. Suggested criteria that might aid in differentiating human-written from AI-generated literature included nuance, style, and originality [10]. Subtle phrasing and word choice might also be giveaways. A rating scale from 1 (very bad) to 5 (very good) was used for each parameter.

Participants were additionally asked whether they thought that the abstract was generated by a newer-generation AI, a more-developed AI, or a human. A short explanation was provided by each participant.

### User Statistics

Descriptive statistics were used to investigate the correlation between the degree of academic experience and the number of correctly identified abstracts on one hand and between the previously mentioned parameters (eg, originality, grammatical soundness) and the correct identification of abstracts on the other. Furthermore, the correlation between the parameters and a researcher’s classification of an abstract was investigated. Interrater reliability was assessed by comparing the assessment of different articles by the same researcher, on the levels of both correct identification and assessed parameters. Intrarater reliability was assessed by comparing the assessments of different evaluators for both previously mentioned parameters.

The Mann-Whitney *U* test, the Wilcoxon *W* test, the *Z* test, and the asymptotic significance (2-tailed) *P* value were determined.

## Results

The results of the analysis are presented in Tables 1-3. Further descriptive statistics are presented in Multimedia Appendix 1.

**Table 1:** The number of human-written and artificial intelligence (AI)–generated texts that were correctly or incorrectly identified by academics with different levels of academic expertise.

Identified role of evaluator	Evaluations of original texts (n=5), n		Evaluations of texts rewritten by AI (n=20), n	
	Human written (correctly identified)	AI–generated	Human written	AI–generated (correctly identified)
Junior orthopedic surgeon	2	3	5	15
Senior orthopedic surgeon	1	4	4	16
Junior qualitative researcher	2	3	8	12
Senior qualitative researcher	4	1	10	10

**Table 2:** This table details how authors judged manuscripts with artificial intelligence (AI)–generated abstracts with respect to whether an advanced large language model or a newer large language model was used.

Identified role of evaluator	Evaluations of advanced AI-generated abstracts (n=10), n			Evaluations of newer unadvanced AI-generated abstracts (n=10), n	
	Advanced AI (correctly identified)	Newer unadvanced AI	Advanced AI		Newer unadvanced AI (correctly identified)
Junior orthopedic surgeon	3	7	5		5
Senior orthopedic surgeon	4	6	6		4
Junior qualitative researcher	3	7	9		1
Senior qualitative researcher	1	9	7		3

**Table 3:** This table represents how artificial intelligence (AI)–detector software judged the articles.

Abstracts	Predicted to be human written, n	Predicted to be AI generated, n
Written by humans	4	1
Rewritten by advanced AI	1	3
Rewritten by newer unadvanced AI	3	2
Completely generated by advanced AI	1	4
Completely generated by newer unadvanced AI	2	3

## Discussion

### Principal Results

The primary results of the study indicate that neither AI-detection software nor human critical appraisal can reliably distinguish AI-generated texts from human-written work. Regarding human detection of AI-generated texts, neither clinical experience nor area of expertise played a role in the evaluation of the presented material. The secondary results of the study indicate that criteria suggested by prior research, such as originality, style, and nuance, did not correlate with whether the researchers identified a text correctly or not. Furthermore, none of the criteria correlated with whether researchers judged a text as human written or AI generated. The qualitative analysis of the written answers did not provide any new insights on the subject in question. However, the junior orthopedic researcher was able to correctly identify texts according to the objectivity parameter. Whether this was due to correct interpretation or chance is unclear. Perhaps future studies with larger sample sizes can help in shedding light on this matter. Selecting the evaluators might have impacted the results of the study. Although the researchers were proficient published authors, English was not their primary language and this might have led to the inability to correctly identify the abstracts. However, the impact of this study is not reduced, as one might argue that scientific literature consumption is not restricted to researchers with English as their mother tongue. Furthermore, reading and publishing in English is becoming common practice, especially if research is considered to be relevant on the international level.

### Comparison With Prior Work

Although AI is an evolutionary technology that presents an enormous potential for future research applications, the results of this study and previous studies with similar methodologies [10] are alarming. AI seems to have reached human-level writing skills, which in combination with its easy accessibility is able to threaten academic integrity. The findings of this analysis contradict previous claims for the ability to detect manuscripts generated by AI through model-agnostic and distribution-agnostic features [19]. Even though nonmalicious applications of AI, including grammatical corrections, reference style adjustment, and thought-process organization, represent plausible uses of AI models, potential fraudulent uses include the generation of complete texts from a simple command. Examples of malicious AI use might also include the rewriting of entire texts [20,21], as shown in this study. AI-generated texts can also be passed through AI-detection software by malicious users, who would then use the texts that passed the examination, making it even more difficult to subsequently detect fraudulent use.

Besides the ability to falsify results, AI presents researchers with the capacity to present false results in a plausible manner [22,23]. This also applies to inaccurate findings being reported confidently, which may be a misrepresentation that could lead to confusion, especially if the results are presented to unexperienced peers. Therefore, fact-checking the AI-generated statements and references will be essential when relying on such tools. AI also the capacity to generate images that can be used in the presentation of results [24]. In the area of orthopedic surgery, AI has already been proven to recognize patterns associated with multiple types of fractures [25]. Combined with its image-generation capacity, AI models will be able to create radiographic representations of fractures that are of no true scientific value but can be used to alter the results of a study.

Additionally, with the ever-increasing human inability to distinguish AI- and human-generated work, new rules must be written to ensure the scientific integrity of every published paper. Suggestions have included an increase in transparency in the design of AI models [26], as well as complete transparency in the use of AI by authors. This includes where and how LLMs were used in scientific projects [8,27].

Understanding the algorithms of these programs might aid in conceiving new and better programs to counteract fraud in its many forms. In an article in the journal *Nature*, the company Turnitin was reported to have incorporate AI-detection software [28].

Finally, and perhaps most importantly, the integrity of research is the most important aspect of the evolving discussion around the use of AI. Many previously conducted cross-examinations of academic publications revealed that research data obtained from prestigious academic institutions and published in equally prestigious academic journals were falsified. Whether these findings were intentionally corrupted or were errors of data collection is of little significance compared to the effects they might have on clinical and academic work. Thus, one can say that AI is just a tool, and its potential to cause good or harm is derived from individual motivations, experience level, and integrity [2]. Calls to completely ban AI from academic endeavors are, in the eyes of the authors, exaggerated, and future fraud can be minimized by optimizing self-regulatory mechanisms [29] and AI-detection models [30,31]. As well as this, the authors of this paper agree that detection of academic fraud is a responsibility of editors and journals, as a letter to *Nature* previously suggested [32]. However, the central role of researchers cannot be overemphasized.

### Limitations

Limitations of this study include the inability to trace AI use in the original articles included in this study. However, we assumed that if AI were used, it would have been reported in the methodology or declarations sections. A second limitation of this study is that English is not the native language of the assessors. However, all the involved researchers have deep levels of proficiency, having published prior research in English. A third limitation is the small sample size of examined individuals and AI-recognition software, which does not allow us to draw definite conclusions on the matter at hand. However, as LLMs in the field of AI become more sophisticated, the recommendations that were made by previous authors and mentioned in this paper will still hold. The final limitation of this study is that a subset of articles dealing with meniscal injuries was chosen from the immense field of orthopedics. This is particularly important when considering the “hot topic” subset.

### Conclusions

The statistical and qualitative analysis of the presented material showed that researchers were unable to differentiate human-written from AI-generated texts. Furthermore, the secondary finding of this study was that previously suggested criteria, such as originality and comprehension, did not aid in the differentiation of human-written and LLM-generated texts. Both findings show that humans and AI-detection software currently fail to properly identify the use of LLMs in the academic literature.

Furthermore, one can only speculate about the amount of undisclosed AI use in the academic literature. However, with the ever-increasing sophistication of LLMs, the integrity of future projects will be entirely dependent on scientists’ attitudes, as AI can serve as a facilitator and accelerator in publishing but can also be used with malicious intent. With regard to replicating this study, the authors strongly recommend that a larger sample size of articles with a larger number of researchers should be considered.

## Appendix

Multimedia Appendix 1 Supplemental tables providing details of the statistical analysis.

## Abbreviations

AI: artificial intelligence
LLM: large language model

## Data Availability

Data will be made available by the corresponding author upon request.
