# Supplementary material for: Human-Written vs AI-Generated Texts in Orthopedic Academic Literature: Comparative Qualitative Analysis
Source: JMIR Form Res. 2024 Feb 16;8:e52164. doi: 10.2196/52164 (PMC10907945; doi:10.2196/52164)
Supplement: Multimedia Appendix 1 [file formative_v8i1e52164_app1.docx]

## Appendix

The tables in the appendix detail the statistical analysis that was conducted to try to correlate the parameters used to differentiate articles with whether articles were correctly identified or not.

| Junior Qualitative Researcher | Gramatical Soundness | Easy Flow | Hot Topic | Comprehension | Structured Writing | Originality |
| --- | --- | --- | --- | --- | --- | --- |
| Mann-Whitney U | 45,000 | 48,000 | 40,500 | 42,000 | 44,000 | 42,500 |
| Wilcoxon W | 255,000 | 258,000 | 250,500 | 57,000 | 254,000 | 252,500 |
| Z | -0,436 | -0,152 | -0,683 | -0,493 | -0,444 | -0,549 |
| Asymp. Sig. (2-tailed) | 0,663 | 0,879 | 0,495 | 0,622 | 0,657 | 0,583 |

| Senior Qualitative Researcher | Gramatical Soundness | Easy Flow | Hot Topic | Comprehension | Structured Writing | Originality |
| --- | --- | --- | --- | --- | --- | --- |
| Mann-Whitney U | 52,000 | 41,500 | 60,000 | 51,500 | 63,000 | 61,000 |
| Wilcoxon W | 88,000 | 77,500 | 196,000 | 187,500 | 199,000 | 97,000 |
| Z | -0,832 | -1,450 | -0,426 | -1,014 | -0,071 | -0,231 |
| Asymp. Sig. (2-tailed) | **0,405** | **0,147** | **0,670** | **0,311** | **0,943** | **0,817** |

| Junior Orthopaedic Surgeon | Gramatical Soundness | Easy Flow | Hot Topic | Comprehension | Structured Writing | Originality |
| --- | --- | --- | --- | --- | --- | --- |
| Mann-Whitney U | 71,500 | 72,000 | 61,000 | 61,000 | 67,500 | 37,000 |
| Wilcoxon W | 116,500 | 117,000 | 197,000 | 106,000 | 112,500 | 173,000 |
| Z | -0,031 | 0,000 | -0,668 | -0,664 | -0,263 | -2,408 |
| Asymp. Sig. (2-tailed) | **0,975** | **1,000** | **0,504** | **0,507** | **0,792** | **0,016** |

| Senior Orthopaedic Surgeon | Gammatical Soundness | Easy Flow | Hot Topic | Comprehension | Structured Writing | Originality |
| --- | --- | --- | --- | --- | --- | --- |
| Mann-Whitney U | 75,500 | 67,500 | 75,500 | 69,500 | 69,500 | 63,000 |
| Wilcoxon W | 141,500 | 133,500 | 141,500 | 174,500 | 135,500 | 168,000 |
| Z | -0,175 | -0,570 | -0,087 | -0,482 | -0,420 | -0,855 |
| Asymp. Sig. (2-tailed) | **0,861** | **0,569** | **0,931** | **0,630** | **0,674** | **0,392** |
